# Synthesis and Biochemical Evaluation of Warhead-Decorated Psoralens as (Immuno)Proteasome Inhibitors

**DOI:** 10.3390/molecules26020356

**Published:** 2021-01-12

**Authors:** Eva Shannon Schiffrer, Matic Proj, Martina Gobec, Luka Rejc, Andrej Šterman, Janez Mravljak, Stanislav Gobec, Izidor Sosič

**Affiliations:** 1 Faculty of Pharmacy, University of Ljubljana, Aškerčeva cesta 7, SI-1000 Ljubljana, Slovenia; eva.shannon.schiffrer@ffa.uni-lj.si (E.S.S.); matic.proj@ffa.uni-lj.si (M.P.); martina.gobec@ffa.uni-lj.si (M.G.); andrej.sterman@ffa.uni-lj.si (A.Š.); janez.mravljak@ffa.uni-lj.si (J.M.); stanislav.gobec@ffa.uni-lj.si (S.G.); 2 Faculty of Chemistry and Chemical Technology, University of Ljubljana, Večna pot 113, 1000 Ljubljana, Slovenia; luka.rejc@fkkt.uni-lj.si

**Keywords:** immunoproteasome, psoralen core, non-peptidic, electrophilic compounds, warhead scan

## Abstract

The immunoproteasome is a multicatalytic protease that is predominantly expressed in cells of hematopoietic origin. Its elevated expression has been associated with autoimmune diseases, various types of cancer, and inflammatory diseases. Selective inhibition of its catalytic activities is therefore a viable approach for the treatment of these diseases. However, the development of immunoproteasome-selective inhibitors with non-peptidic scaffolds remains a challenging task. We previously reported 7*H*-furo[3,2-*g*]chromen-7-one (psoralen)-based compounds with an oxathiazolone warhead as selective inhibitors of the chymotrypsin-like (β5i) subunit of immunoproteasome. Here, we describe the influence of the electrophilic warhead variations at position 3 of the psoralen core on the inhibitory potencies. Despite mapping the chemical space with different warheads, all compounds showed decreased inhibition of the β5i subunit of immunoproteasome in comparison to the parent oxathiazolone-based compound. Although suboptimal, these results provide crucial information about structure–activity relationships that will serve as guidance for the further design of (immuno)proteasome inhibitors.

## 1. Introduction

In mammals, most intracellular proteins are destined for degradation, which involves the proteasome, a multiprotease complex [1,2,3]. The 26S proteasome represents the heart of the ubiquitin-proteasome system that is responsible for the maintenance of protein homeostasis and the regulation of various cellular processes [4,5,6]. It is a nucleophilic hydrolase with *N*-terminal Thr1 acting as a nucleophile to cleave the peptide bond of proteins [7]. The 26S proteasome is comprised of a 20S core particle (CP) and 19S regulatory units. The 20S core is a 720 kDa large barrel-shaped structure assembled of four stacked rings, each consisting of seven subunits. The two outer α rings provide structural integrity and act like “gates” allowing the entry of unfolded proteins to the two inner β rings, which contain three catalytically active subunits responsible for proteolysis of substrates [8]. Subunit β1 shows caspase-like activity, subunit β2 trypsin-like activity, whereas subunit β5 exhibits chymotrypsin-like activity [9,10]. There are three individual CP types: the constitutive proteasome (cCP), which is expressed in all eukaryotic cells, the thymoproteasome (tCP) [11], which is exclusive to cortical thymic epithelial cells, and the immunoproteasome (iCP) [12], which is expressed in cells of hematopoietic origin, but can also be induced in other tissues. Namely, the induction of iCP in other cell types is possible during acute immune and inflammatory responses [13,14,15]. Exposure to inflammatory factors, such as tumor necrosis factor α and interferon-γ causes the expression of the iCP active subunits β (designated as β1i, β2i, β5i), which replace their constitutive counterparts [12,16].

Increased expression of cCP and iCP can lead to a number of diseases. These include many types of cancer, infections, inflammatory and autoimmune diseases (Crohn’s disease, ulcerative colitis, hepatitis, and rheumatoid arthritis), as well as neurological disorders [17,18,19,20,21,22,23]. The cCP and the iCP therefore represent validated targets for the design of new pharmacologically active compounds [24,25,26,27]. The druggability of both CPs is clearly represented by the clinically used covalent inhibitors bortezomib, carfilzomib, and ixazomib, which are used for the treatment of multiple myeloma and mantle-cell-lymphoma [27]. Selective inhibition of the iCP’s β5i [28] subunit or simultaneously acting on β1i and β5i catalytic activities [29,30] are both approaches that are being investigated in the treatment of autoimmune and inflammatory diseases. In addition, such strategy should cause fewer adverse effects, as the expression of iCP is induced during the course of disease processes [31,32]. By avoiding cCP inhibition, the protein degradation would thus not be inhibited in most eukaryotic cells.

**Figure 1:**
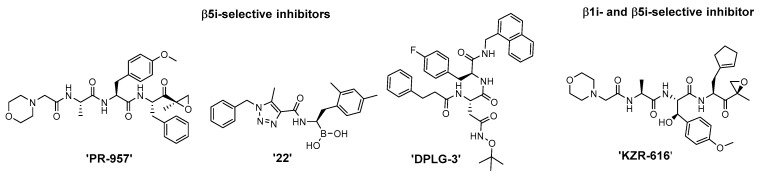
Structures of the most studied iCP-selective peptidic inhibitors. For a more thorough overview on subunit-selective iCP inhibitors, the reader is referred to recent reviews [32,35].

The most advanced iCP inhibitors that are frequently utilized in functional studies of iCP inhibition are represented in Figure 1. Please note that only a selected number of derivatives is depicted; namely, the most studied β5i-selective inhibitor PR-957 [28], β1i and β5i dual inhibitors KZR-616 [29] and ‘compound 22′ [33], as well as the most selective β5i inhibitor DPLG-3 [34]. Structurally, these compounds all possess a peptidic backbone. Moreover, the former three compounds are all endowed with an electrophilic warhead, which reacts with the catalytic Thr1 of the proteasome subunits to form a covalent bond and to confer improved inhibition [24].

**Figure 2:**
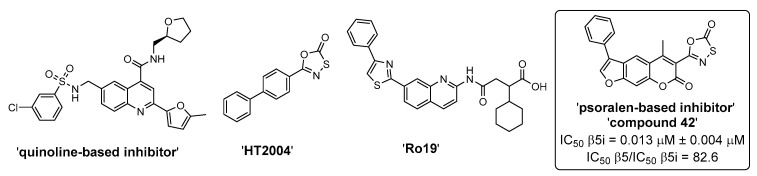
A selection of non-peptidic iCP inhibitors. ‘Compound 42’ [39] was the most selective irreversible β5i subunit inhibitor from the initial series of psoralen-based inhibitors. It represents the parent compound for studies in this manuscript.

Because peptidic compounds, such as bortezomib and carfilzomib, are prone to poor metabolic stability and low bioavailability due to the unfavorable physico-chemical characteristics [36,37,38], there is a need to develop inhibitors with non-peptidic scaffolds. Despite being significantly less represented, there were some recent reports on non-peptidic inhibitors of the iCP (mostly inhibiting the β5i subunit) and the representative compounds are shown in Figure 2 [39,40,41,42,43,44]. As with peptidic compounds, irreversible inhibitors of non-peptidic nature can be obtained through structure-guided optimization, whereby an electrophilic warhead is properly positioned onto the structure of the non-covalently binding scaffold [45]. An essential prerequisite for this strategy to work is that the position of the electrophilic moiety allows the formation of the covalent bond between the inhibitor and the catalytic Thr1.

**Figure 3:**
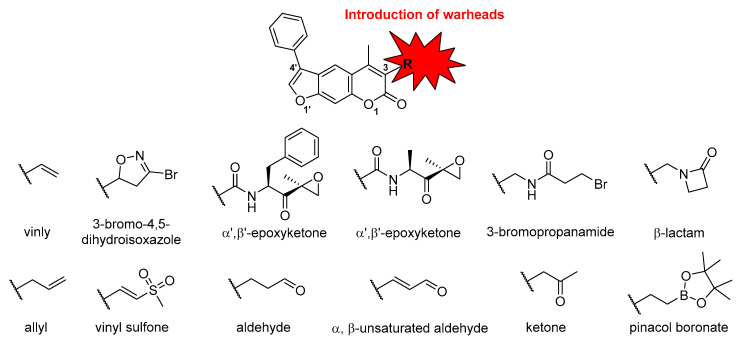
Schematic representation of the work described in this study. The numbering system for the psoralen ring is shown for clarity, as well as general nomenclature for the warhead moieties used.

Recently, we discovered non-peptidic and β5i-selective inhibitors with a central psoralen core [39]. The most potent non-covalent inhibitor obtained during structure-activity relationship (SAR) studies possessed a phenyl substituent at position 4′ (see Figure 3 for psoralen atom numbering). This compound was also transformed into two potent irreversible covalent inhibitors by adding electrophilic warheads at position 3, i.e., succinimidyl ester and oxathiazolone. Of these two compounds, the oxathiazolone-based inhibitor showed the most promising inhibitory characteristics (Figure 2, ‘compound 42′) as it was a potent and selective iCP inhibitor [39]. It was demonstrated previously that oxathiazolones inhibit iCP via cyclocarbonylation of the β-OH and α-NH_2_ of the active site Thr1 [41]. Nevertheless, this structural fragment is deemed hydrolytically unstable making it less suitable for further development [41]. This fact prompted us to investigate other possible warheads that could be attached at the same position of the psoralen core. Previously, we already determined that acrylamides and nitrile-based warheads led to worse inhibition of the iCP [39]. However, to further map the warhead chemical space attached onto the psoralen core, we prepared a new focused set of compounds with different electrophilic fragments attached at position 3 (Figure 3), and evaluated their influence on the inhibition of all six catalytic subunits of both CPs. The selection of warheads in this study was based both on previously well described Thr targeting warheads (e.g., vinyl sulfones, α’,β’-epoxyketones) [24], as well as on biologically less represented electrophilic moieties. In addition, to minimize the influence of non-covalently binding portion of the molecule on overall inhibitory potency, we used the same core compound with a phenyl substituent at position 4′.

## 2. Results and Discussion

### 2.1. Syntheses of 3-Substituted Psoralens

**Scheme 1:**
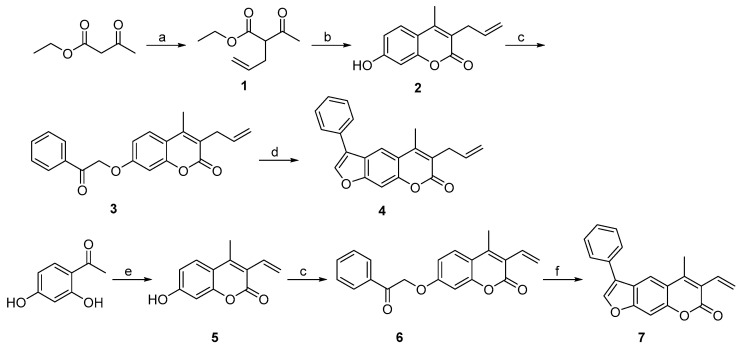
Synthesis of compounds with allyl (**4**) and vinyl (**7**) warheads attached at position 3 of the psoralen ring. Reagents and conditions: (a) allyl bromide, NaH (60%), THF, 0 °C to rt, overnight; (b) resorcinol, 98% H_2_SO_4_, dioxane, 0 °C to rt, overnight; (c) 2-bromoacetophenone, K_2_CO_3_, KI, dioxane, 100 °C, 24 h; (d) 1 M NaOH, propan-2-ol, 80 °C, 40 min; (e) crotonyl chloride, K_2_CO_3_, acetone, 60 °C, 24 h; (f) 1 M KOH, EtOH, 85 °C, 2 h.

To prepare 3-allyl-substituted psoralen, ethyl acetoacetate was used as a starting material (Scheme 1). It was first alkylated using NaH as a base to obtain compound **1**, which was subjected to Pechmann reaction conditions to yield 7-hydroxycoumarin derivative **2**. After OH group alkylation with 2-bromoacetophenone, the final allyl-substituted compound **4** was obtained by base-catalyzed condensation of the coumarin derivative **3** into psoralen ring (Scheme 1). A compound with 3-vinyl-based warhead attached at position 3 (compound **7**) was obtained via a similar route. The crucial intermediate 7-hydroxy-4-methyl-4-vinyl-2*H*-chromen-2-one (**5**) was obtained in high yield by heating resorcinol derivative and crotonyl chloride at 60 °C in acetone. This was followed by a 2-bromoacetophenone-mediated alkylation and cyclization into psoralen yielding compounds **6** and **7**, respectively.

**Scheme 2:**
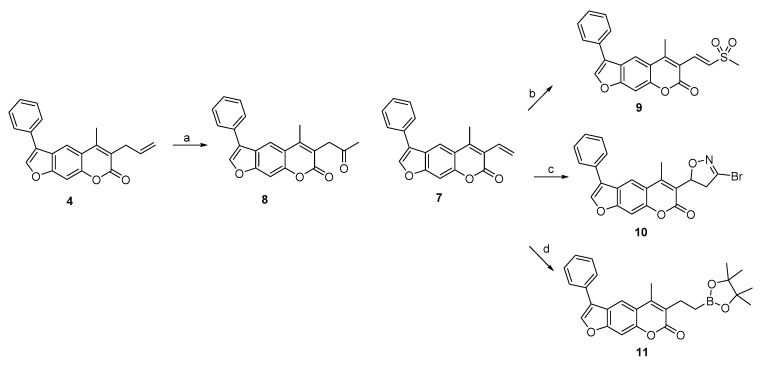
Synthesis of compounds **8**, **9**, **10**, and **11** with ketone, vinyl sulfone, 3-bromo-4,5-dihydroisoxazole, and pinacolate ester, respectively, as warheads. Reagents and conditions: (a) Dess–Martin periodinane, Pd(OAc)_2_, CH_3_CN, H_2_O, 50 °C, overnight; (b) DMSO, H_2_O, NH_4_I, 130 °C, 36 h; (c) 1,1-dibromoformaldoxime, DMF, NaHCO_3_, −15 °C to rt, 5 h; (d) bis(pinacolato)diboron, CsF, 1,4-dioxane, MeOH, 100 °C, 12 h.

Compounds **4** and **7** were further used as synthons to prepare derivatives with other electrophilic moieties at position 3 (Scheme 2). The former was used in a Wacker-type oxidation of the terminal olefin by the combination of Pd(OAc)_2_ and Dess-Martin periodinane to prepare the compound with ethyl methyl ketone moiety, i.e., compound **8**. The vinyl-substituted derivative **7** was a starting point for three different warhead-decorated psoralens, namely vinyl sulfone **9** (via NH_4_I-induced sulfonylation of vinyl at position 3 with DMSO), 3-bromo-4,5-dihydroisoxazole **10** [46] (via cycloaddition of the alkene with 1,1,-dibromoformaldoxime), and pinacolate ester **11** (via transition-metal-free synthesis of alkylboronate from vinyl and bis(pinacolato)diboron) (Scheme 2).

**Scheme 3:**
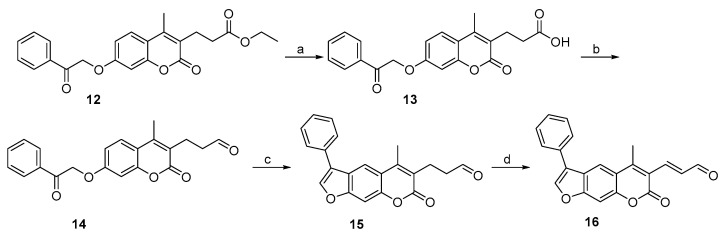
Synthesis of compounds with aldehyde- (**15**) and α,β-unsaturated aldehyde-based (**16**) warheads attached at position 3 of the psoralen ring. Reagents and conditions: (a) 1 M HCl, dioxane, reflux, 2 h; (b) i. SOCl_2_, DMF, toluene, rt, 17 h; ii. H_2_, Pd/BaSO_4_, toluene, 100 °C, 2 h; (c) 1 M NaOH, propan-2-ol, 60 °C, 15 min; (d) SeO_2_, dioxane, H_2_O, MW, 150 °C, 1 h. Synthesis of compound **12** was described previously [39].

**Figure 4:**
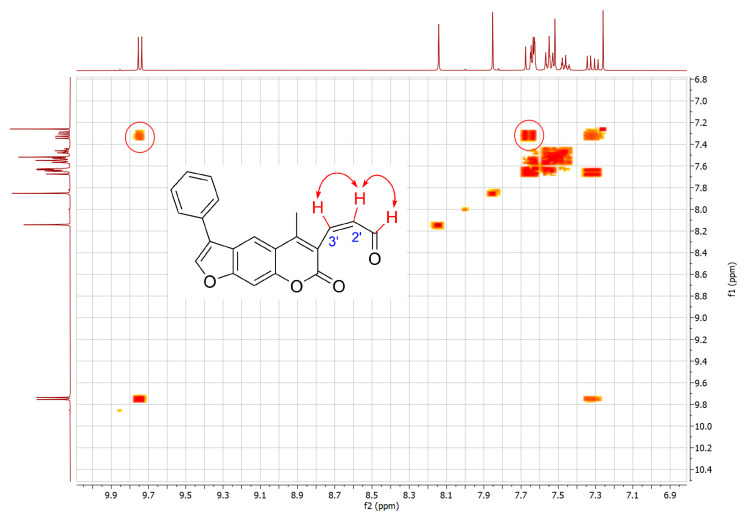
COSY experiment for **16**. Circled cross-peaks indicate coupling between aldehyde proton CHO and the adjacent C2′-H, and between C2′-H and C3′-H.

**Figure 5:**
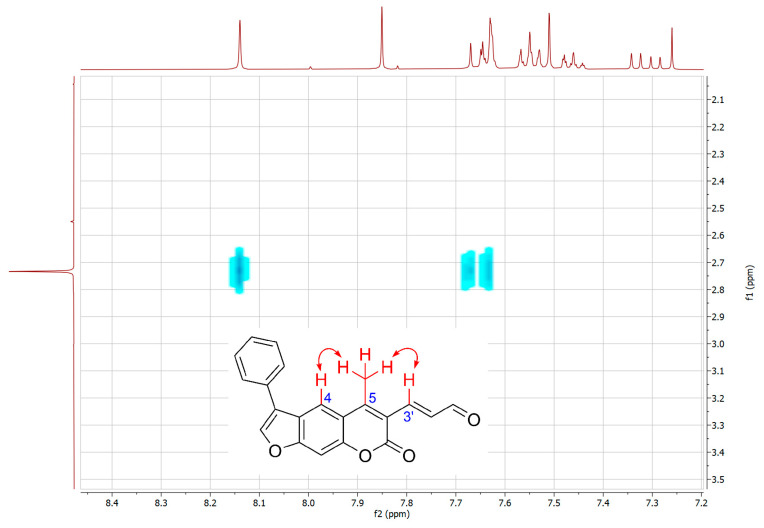
NOESY experiment for **16**. Only cross-peaks that indicate coupling between CH_3_ protons and C4-H and C3′-H are shown.

The synthesis of 3-propanal-substituted psoralen **15** was initiated by a coumarin derivative **12** possessing ethyl propionate moiety at position 3 (Scheme 3). The acidic hydrolysis yielded propanoic acid **13**, which was transformed into aldehyde derivative **14** by first forming an acid chloride, followed by in situ reduction with hydrogen gas using Pd/BaSO_4_ as a catalyst. Interestingly, an attempt to prepare α-ketoaldehyde (which is a known Thr-targeting warhead [24]) from compound **15** by Riley oxidation with SeO_2_ resulted in the formation of α,β-unsaturated aldehyde derivative **16** (Scheme 3, Figure 4 and Figure 5).

To further confirm the structure of α,β-unsaturated aldehyde **16**, two-dimensional NMR experiments correlation spectroscopy (COSY) and Nuclear Overhauser effect spectroscopy (NOESY) were recorded. In the COSY spectrum (Figure 4), a clear correlation between the aldehyde proton CHO and the adjacent C2′-H was observed. In addition, the NOESY experiment showed a coupling between the CH_3_ protons and C4-H and C3′-H (Figure 5).

**Scheme 4:**
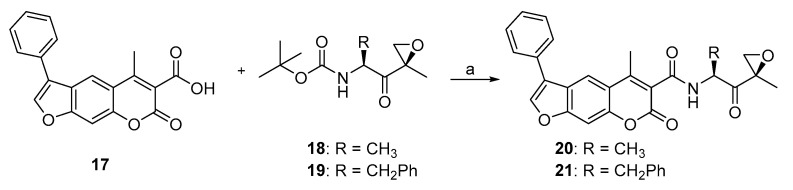
Synthesis of epoxyketone-based compounds **20** and **21**. Reagents and conditions: (a) i. TFA, CH_2_Cl_2_, 0 °C, 30 min; ii. HATU, HOBt×H_2_O, DIPEA, DMF (**20**) or CH_2_Cl_2_ (for **21**), rt, 24 h. Syntheses of compounds **17**, **18**, and **19** were based on previously described procedures [39], compound **17**; [47], compound **18**; [48], compound **19**. All spectral data (^1^H-NMR, HRMS) corresponded well to the original reports.

The fact that the most advanced selective iCP inhibitors and also carfilzomib, which is a marketed cCP and iCP inhibitor, possess an α’,β’-epoxyketone fragment as the Thr-targeting warhead, encouraged us to prepare two such psoralen-based compounds (Scheme 4). Both **20** and **21** were synthesized from the corresponding precursors **17**, **18**, and **19** by a HATU-mediated amide bond formation (Scheme 4).

**Scheme 5:**
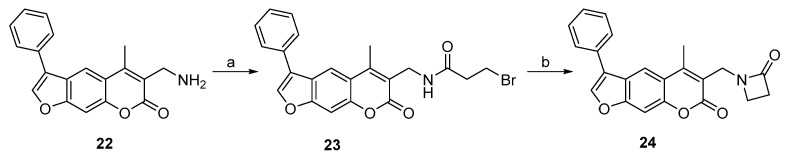
Synthesis of alkyl bromide-based psoralen **23** and psoralen **24** with azetidin-2-one as a warhead. Reagents and conditions: (a) 3-bromopropanoyl chloride, K_2_CO_3_, CH_2_Cl_2_, 0 °C to rt, 3 h; (b) NaO*t*Bu, DMF, 0 °C to rt, 24 h. Synthesis of compound **22** was described previously [39].

To prepare 3-azetidin-2-one-substituted psoralen **24**, a previously synthesized compound **22** was used as a crucial intermediate. It was first *N*-acylated with 3-bromopropanoyl chloride to yield 3-bromopropanamide **23**, and then cyclized into the β-lactam ring by using NaO*t*Bu as a base (Scheme 5).

### 2.2. Biochemical Evaluation

The target compounds were evaluated for their inhibitory potencies on both CPs (Table 1) using subunit selective fluorogenic substrates (for details, see Materials and Methods Section). The data were calculated as residual activities (RAs) of individual subunits of CPs in the presence of 1 µM of each compound. This concentration was used due to poor solubility of all final compounds at higher concentrations, emphasizing the need for development of inhibitors with improved solubility. The previously described oxathiazolone derivative ‘compound 42′ and carfilzomib were used as positive control using the same concentration (1 µM) to enable a better comparison between compounds.

**Figure 6:**
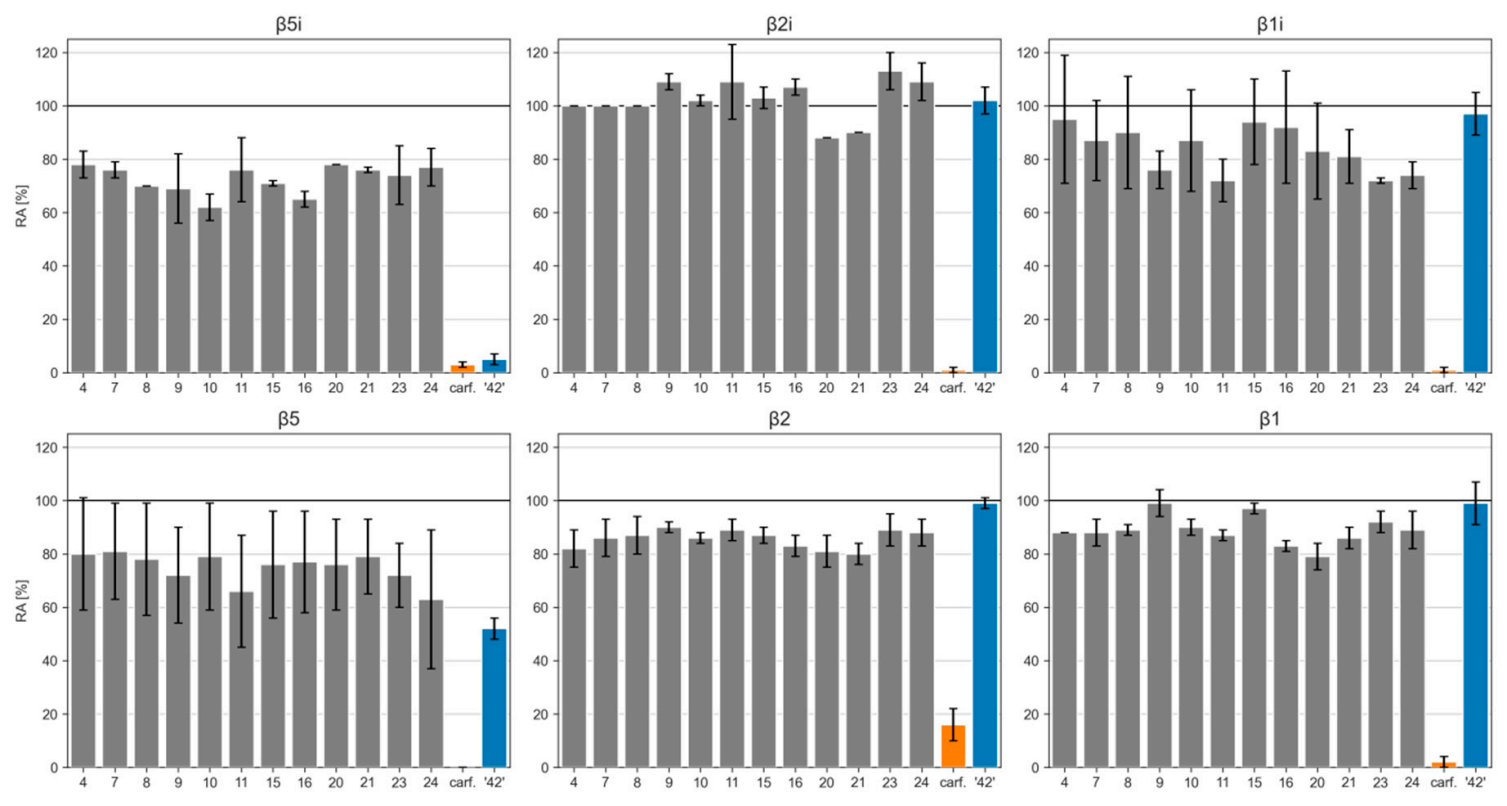
Inhibition results represented as bar charts of inhibition percentage. carf.: carfilzomib.

**Figure 7:**
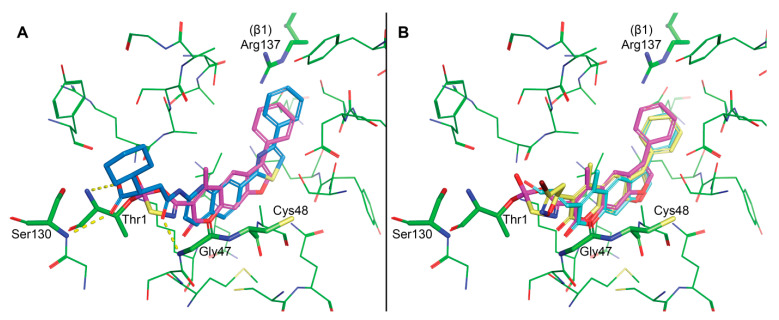
Molecular modelling. Binding site residues are presented as green sticks with labels for some of the key residues. (**A**) Covalent docking of ‘42′ (magenta) into the β5i subunit (PDB: 5M2B). Please note that only the initial intermediate formed after the nucleophilic attack of OH group of Thr1 onto the carbonyl group of the oxathiazolone is represented. Co-crystalized ligand Ro19 is presented with blue sticks and dashed yellow lines for hydrogen bonds. (**B**) Noncovalent docking of **10** (cyan) and **16** (yellow) reveals good alignment of the psoralen core with the proposed pose of ‘42′ (magenta). However, the distance from the electrophilic carbons of **10** and **16** to the catalytic Thr1O^γ^ is too large to form a covalent bond.

Given the fact that all assayed compounds possessed the same non-covalently binding portion, we were able to thoroughly assess the contributions of attached warheads to the inhibition of all catalytically active subunits of iCP and cCP. The assay results showed that all new psoralens were worse inhibitors of β5i subunit of iCP in comparison to the parent oxathiazolone-based ‘compound 42′ (Table 1, Figure 6). This is most probably due to the mispositioning of the electrophilic carbons of all compounds and the catalytic Thr1O*^γ^* in the β5i active site. Interestingly, all compounds inhibited β5i activity with a similar potency at 1 µM with RA values ranging from 62 to 78%. Of the 12 prepared compounds, 3-bromo-4,5-dihydroisoxazole-substituted psoralen **10** and compound **16** with an α,β-unsaturated aldehyde as the warhead were the most promising. The former showed RA value of 62 ± 5%, whereas for the latter RA was determined at 65 ± 3% (see also postulated binding modes for 10 and 16 in Figure 7). It was not surprising to see that all 12 compounds also exhibited worse inhibition of the β5 subunit of cCP, albeit these differences were much less pronounced as for the β5i subunit. Of note, compounds **9**, **11**, **23**, and **24** were slightly better inhibitors of β1i subunit of iCP in comparison to the ‘compound 42′. All psoralen-based compounds (with oxathiazolone included) did not inhibit other subunits of both CPs (i.e., β2i, β2, and β1), whereas carfilzomib completely abolished activity of all subunits at 1 µM (Table 1, Figure 6).

## 3. Materials and Methods

### 3.1. General Chemistry Methods

Reagents and solvents were obtained from commercial sources (Acros Organics (Thermo Fisher Scientific, Waltham, MA, USA), Sigma-Aldrich (Merck KGaA, Darmstadt, Germany), TCI Europe (Tokyo Chemical Industry, Tokyo, Japan), Alfa Aesar (Thermo Fisher Scientific, Waltham, MA, USA), Fluorochem (Fluorochem Ltd., Derbyshire, UK) and were used as received. Carfilzomib were purchased from MedChemExpress. For reactions involving air or moisture sensitive reagents, solvents were distilled before use and these reactions were carried out under nitrogen or argon atmosphere. Reactions using microwaves were performed on a standard monomode microwave reactor MONOWAVE 200 (Anton Paar, Graz, Austria). Reactions were monitored using analytical thin-layer chromatography plates (Merck 60 F254, 0.20 mm), and the components were visualized under UV light and/or through staining with the relevant reagent. Normal phase flash column chromatography was performed on Merck Silica Gel 60 (particle size 0.040–0.063 mm; Merck, Germany). ^1^H and ^13^C-NMR spectra were recorded at 295 K on a Bruker Avance III 400 MHz spectrometer (Bruker, Billerica, MA, USA) operating at frequencies for ^1^H-NMR at 400 MHz and for ^13^C-NMR at 101 MHz. The chemical shifts (δ) are reported in parts per million (ppm) and are referenced to the deuterated solvent used. The coupling constants (*J*) are given in Hz, and the splitting patterns are designated as follows: s, singlet; br s, broad singlet; d, doublet; app d, apparent doublet; dd, doublet of doublets; ddd, doublet of doublets of doublets; t, triplet; dt, doublet of triplets; td, triplet of doublets; m, multiplet. All ^13^C-NMR spectra were proton decoupled. Mass spectra data and high-resolution mass measurements were performed on a Thermo Scientific Q Exactive Plus Hybrid Quadrupole-Orbitrap mass spectrometer (Thermo Fisher Scientific, Waltham, MA, USA). The purity of the compounds used in biochemical assays was determined with analytical normal-phase HPLC on an Agilent 1100 LC modular system (Agilent, Santa Clara, CA, USA) that was equipped with a photodiode array detector set to 254 nm. A Kromasil 3-CelluCoat column (150 mm × 4.6 mm; 3 μm particle size) was used, with a flow rate of 1.0 mL/min and a sample injection volume of 5–20 µL. An isocratic eluent system of A (hexane) and B (isopropanol) was used; the ratio used is described for each compound below. The purities of the test compounds used for the biological evaluations were ≥95%, unless stated otherwise.

### 3.2. Syntheses

Synthesis of ethyl 2-acetylpent-4-enoate (**1**):

To a solution of ethyl acetoacetate (7.28 mL, 7.50 g, 57.60 mmol, 1 equiv.) in 50 mL of anhydrous THF, NaH in mineral oil (60%, 2.30 g, 57.60 mmol, 1 equiv.) was added and the resulting suspension stirred under argon at 0 °C. After 20 min, a solution of allyl bromide (4.99 mL, 6.97 g, 57.60 mmol, 1 equiv.) in 25 mL of anhydrous THF was added dropwise. The reaction mixture was stirred at room temperature overnight. Next, cold H_2_O (25 mL) was added and THF was evaporated under reduced pressure. The resulting suspension was extracted with Et_2_O (3 × 25 mL), the organic layer separated, dried over anhydrous Na_2_SO_4_, and evaporated. The product was purified by column chromatography (Et_2_O/petroleum ether, 1/5, *v*/*v*). Yield: 71%, clear liquid. ^1^H-NMR (400 MHz, DMSO-*d*_6_) δ 1.17 (t, *J* = 7.1 Hz, 3H, CH_3_CH_2_), 2.18 (s, 3H, COCH_3_), 2.42–2.47 (m, 2H, CH_2_CHCH_2_CH), 3.73 (dd, *J* = 7.8, 6.8 Hz, 1H, CH), 4.07–4.15 (qd, 2H, *J* = 7.1, 1.6 Hz, CH_3_CH_2_), 4.98–5.10 (m, 2H, CH_2_CHCH_2_CH), 5.67–5.77 (m, 1H, CH_2_CHCH_2_CH).

Synthesis of 3-allyl-7-hydroxy-4-methyl-2*H*-chromen-2-one (**2**):

This compound was prepared using Pechmann condensation as follows. A solution of resorcinol (4.06 g, 36.90 mmol, 1 equiv.) and ethyl 2-acetylpent-4-enoate (**1**) (6.90 g, 40.50 mmol, 1.1 equiv.) in dioxane (80 mL) was cooled to 0 °C, followed by drop-wise addition of concentrated H_2_SO_4_ (98%, 19.60 mL, 405 mmol, 10 equiv.). The reaction mixture was stirred at room temperature overnight. Dioxane was then evaporated under reduced pressure and the semi-solid mixture was added portion-wise to an ice-cold solution of KOH (40 g) in H_2_O (100 mL). The pH was adjusted to 13 with KOH and the resulting white solid was filtered off. The filtrate was extracted with EtOAc (3 × 25 mL) and the combined organic extracts were dried over anhydrous Na_2_SO_4_ and evaporated under reduced pressure. The compound was purified by column chromatography (EtOAc/*n*-hexane, 1/1.5, *v*/*v*, dry loading) yielding a pale-yellow solid. Yield: 13%. ^1^H-NMR (400 MHz, DMSO-*d*_6_) δ 2.34 (s, 3H, CH_3_), 3.30 (d, *J* = 6.0 Hz, 2H, Ar-CH_2_CHCH_2_), 4.98–5.06 (m, 2H, Ar-CH_2_CHCH_2_), 5.84 (ddt, *J* = 16.3, 10.3, 6.0 Hz, 1H, Ar-CH_2_CHCH_2_), 6.70 (d, *J* = 2.4 Hz, 1H, Ar-H), 6.80 (dd, *J* = 8.7, 2.4 Hz, 1H, Ar-H), 7.63 (d, *J* = 8.7 Hz, 1H, Ar-H), 10.44 (s, 1H, OH); HRMS (ESI) *m/z* calculated for C_13_H_11_O_3_ [M − H]^−^ 215.0714, found 215.0707.

Synthesis of 3-allyl-4-methyl-7-(2-oxo-2-phenylethoxy)-2*H*-chromen-2-one (**3**):

This compound was synthesized following a previously described procedure [39]. Briefly, to a solution of 3-allyl-7-hydroxy-4-methyl-2*H*-chromen-2-one (**2**) (0.99 g, 4.56 mmol, 1 equiv.) in dioxane (70 mL), K_2_CO_3_ (2.52 g, 18.22 mmol, 4 equiv.) and KI (76 mg, 0.46 mmol, 0.1 equiv.) were added. After 10 min of stirring at 100 °C, 2-bromoacetophenone (1.36 g, 6.83 mmol, 1.5 equiv.) was added and the mixture was further stirred at 100 °C for 24 h. The solvent was then removed under reduced pressure, followed by addition of H_2_O (30 mL) to the residue. The aqueous phase was extracted with EtOAc (3 × 30 mL), the combined organic extracts were washed with brine (20 mL), dried over anhydrous Na_2_SO_4_, filtered, and evaporated under reduced pressure. The compound was purified by crystallization from MeOH yielding pale-yellow crystalline solid. Yield: 67%. ^1^H-NMR (400 MHz, DMSO-*d*_6_) δ 2.38 (s, 3H, CH_3_), 3.32 (d, *J* = 6.0 Hz, 2H, Ar-CH_2_CHCH_2_), 5.03 (ddd, *J* = 8.5, 3.0, 1.3 Hz, 2H, Ar-CH_2_CHCH_2_), 5.75 (s, 2H, CH_2_), 5.86 (ddt, *J* = 16.2, 10.2, 6.0 Hz, 1H, Ar-CH_2_CHCH_2_), 7.03 (dd, *J* = 8.9, 2.6 Hz, 1H, Ar-H), 7.07 (d, *J* = 2.5 Hz, 1H, Ar-H), 7.59 (app dd, *J* = 10.6, 4.8 Hz, 2H, 2 × Ar-H), 7.68–7.77 (m, 2H, 2 × Ar-H), 8.04 (app dd, *J* = 8.4, 1.2 Hz, 2H, 2 × Ar-H); HRMS (ESI) *m/z* calculated for C_21_H_19_O_4_ [M + H]^+^ 335.1280, found 335.1272.

Synthesis of 6-allyl-5-methyl-3-phenyl-7*H*-furo[3,2-*g*]chromen-7-one (**4**):

This compound was synthesized following a previously described procedure [39]. Namely, to a heated (80 °C) and stirred solution of **3** (0.25 g, 0.75 mmol, 1 equiv.) in propan-2-ol (25 mL), an aqueous solution of NaOH (7.5 mL, 1 M, 10 equiv.) was added. The reaction mixture was stirred at 80 °C for 40 min. After the reaction was complete (monitored by TLC), propan-2-ol was evaporated under reduced pressure. The aqueous residue was acidified with HCl (6 mL, 1 M) to pH 5, then H_2_O (20 mL) was added, the aqueous layer was extracted with CH_2_Cl_2_ (3 × 25 mL), and the combined organic extracts were evaporated under reduced pressure. The compound was purified by column chromatography (Et_2_O/petroleum ether, 1/3, *v*/*v*). White solid, yield: 70%. ^1^H-NMR (400 MHz, DMSO-*d_6_*) δ 2.54 (s, 3H, CH_3_), 3.40 (d, *J* = 6.0 Hz, 2H, Ar-CH_2_CHCH_2_), 5.01–5.11 (m, 2H, Ar-CH_2_CHCH_2_), 5.89 (ddt, *J* = 16.1, 10.2, 6.0 Hz, 1H, Ar-CH_2_CHCH_2_), 7.41–7.48 (m, 1H, Ar-H), 7.51–7.60 (m, 2H, 2 × Ar-H), 7.79 (s, 1H, Ar-H), 7.80–7.82 (m, 1H, Ar-H), 7.83 (t, *J* = 1.6 Hz, 1H, Ar-H), 8.18 (s, 1H, Ar-H), 8.48 (s, 1H, Ar-H); ^1^H-NMR (400 MHz, CDCl_3_) δ 2.49 (s, 3H, CH_3_), 3.49 (d, *J* = 6.0 Hz, 2H, Ar-CH_2_CHCH_2_), 5.01–5.16 (m, 2H, Ar-CH_2_CHCH_2_), 5.94 (ddt, *J* = 16.2, 10.1, 6.0 Hz, 1H, Ar-CH_2_CHCH_2_), 7.44 (ddd, *J* = 7.4, 4.0, 1.3 Hz, 1H, Ar-H), 7.49–7.51 (m, 1H, Ar-H), 7.51–7.57 (m, 2H, 2 × Ar-H), 7.61–7.64 (m, 1H, Ar-H), 7.65 (t, *J* = 1.7 Hz, 1H, Ar-H), 7.82 (s, 1H, Ar-H), 8.00 (s, 1H, Ar-H); ^13^C-NMR (101 MHz, DMSO-*d*_6_) δ 15.19, 30.94, 99.48, 115.69, 116.46, 116.99, 121.18, 121.29, 122.81, 127.23, 127.84, 129.22, 130.72, 134.50, 144.29, 148.39, 149.94, 155.92, 160.58; HRMS (ESI) *m/z* calculated for C_21_H_17_O_3_ [M + H]^+^ 317.1172, found 317.1166. Purity by HPLC (0–18 min; 70% *n*-hexane/isopropanol): 99%.

Synthesis of 7-hydroxy-4-methyl-4-vinyl-2*H*-chromen-2-one (**5**):

A suspension of 1-(2,4-dihydroxyphenyl)ethan-1-one (502 mg, 3.3 mmol, 1 equiv.), crotonyl chloride (395 µL, 429 mg, 4.1 mmol, 1.25 equiv.) and K_2_CO_3_ (1.47 g, 10.6 mmol, 3.2 equiv.) in acetone (25 mL) was heated at 60 °C for 24 h. The solvent was then evaporated under reduced pressure, followed by the addition of EtOAc (100 mL). The organic phase was extracted with H_2_O (100 mL), and the aqueous phase acidified with 2 M HCl and further extracted with EtOAc (2 × 100 mL). The combined organic extracts were dried over anhydrous Na_2_SO_4_, filtered, and evaporated under reduced pressure. The compound was purified by column chromatography (EtOAc/*n*-hexane, 1/4, *v*/*v*). White solid, yield: 71%. ^1^H-NMR (400 MHz, DMSO-*d*_6_) δ 2.45 (s, 3H, CH_3_), 5.51 (dd, *J* = 12.0 Hz, 2.4 Hz, 1H, Ar-CHCH_2_), 6.02 (dd, *J* = 17.4 Hz, 2.4 Hz, 1H, Ar-CHCH_2_), 6.67 (d, *J* = 2.4 Hz, 1H, Ar-H), 6.72 (dd, *J* = 17.4, 12.0 Hz, 1H, Ar-CHCH_2_), 6.79 (d, *J* = 8.9, 2.4 Hz, 1H, Ar-H), 7.66 (d, *J* = 8.9 Hz, 1H, Ar-H), 10.51 (br s, 1H, OH). HRMS (ESI) *m/z* calculated for C_12_H_9_O_3_ [M − H]^−^ 201.0557, found 201.0549.

Synthesis of 4-methyl-7-(2-oxo-2-phenylethoxy)-3-vinyl-2*H*-chromen-2-one (**6**):

This compound was synthesized following a previously described procedure [39]; using the procedure as for **5**. The compound was purified by crystallization from EtOH yielding off-white crystalline solid. Yield: 79%. ^1^H-NMR (400 MHz, CDCl_3_) δ 2.48 (s, 3H, CH_3_), 5.38 (s, 2H, OCH_2_), 5.63 (dd, *J* = 11.8, 1.9 Hz, 1H, Ar-CH=CH_2_), 6.04 (dd, *J* = 17.6, 1.9 Hz, 1H, Ar-CH=CH_2_), 6.71 (dd, *J* = 17.6, 11.8 Hz, 1H, Ar-CH=CH_2_), 6.77 (d, *J* = 2.6 Hz, 1H, Ar-H), 6.96 (dd, *J* = 9.0, 2.6 Hz, 1H, Ar-H), 7.49–7.57 (m, 2H, Ar-H), 7.59 (d, *J* = 8.9 Hz, 1H, Ar-H), 7.70–7.63 (m, 1H, Ar-H), 7.95–8.04 (m, 2H, 2 × Ar-H); ^13^C-NMR (101 MHz, CDCl_3_) δ 15.37, 70.55, 101.43, 112.79, 114.88, 120.10, 122.26, 126.34, 127.99, 128.99, 129.03, 134.14, 134.26, 146.69, 153.55, 160.18, 160.39, 193.18; HRMS (ESI) *m/z* calculated for C_20_H_17_O_4_ [M + H]^+^ 321.1121, found 321.1123.

Synthesis of 5-methyl-3-phenyl-6-vinyl-7*H*-furo[3,2-*g*]chromen-7-one (**7**):

To a solution of 4-methyl-7-(2-oxo-2-phenylethoxy)-3-vinyl-2*H*-chromen-2-one (**6**) (132 mg, 0.4 mmol, 1 equiv.) in EtOH (5 mL), KOH (1.2 mL, 1 M, 1.2 mmol, 3 equiv.) was added and the reaction mixture stirred at 85 °C for 2 h. The solvent was then evaporated, followed by the addition of H_2_O (20 mL). The suspension was acidified with concentrated HCl to pH = 1 and extracted with CH_2_Cl_2_ (2 × 50 mL). The combined organic extracts were dried over Na_2_SO_4_, filtered, and evaporated under reduced pressure. The product was purified by column chromatography (EtOAc/*n*-hexane, 1/1, *v*/*v*). Yellow solid; yield: 78 %; ^1^H-NMR (400 MHz, CDCl_3_) δ 2.60 (s, 3H, CH_3_), 5.69 (dd, *J* = 11.8 Hz, 1.8 Hz, 1H, Ar-CH=CH_2_), 6.05 (dd, *J* = 17.7 Hz, 1.8 Hz, 1H, Ar-CH=CH_2_), 6.77 (dd, *J* = 17.7, 11.8, 1H, Ar-CH=CH_2_), 7.42–7.47 (m, 1H, Ar-H), 7.49 (s, 1H, Ar-H), 7.51–7.56 (m, 2H, Ar-H), 7.62–7.66 (m, 2H, Ar-H), 7.82 (s, 1H, Ar-H), 8.04 (s, 1H, Ar-H); ^13^C-NMR (101 MHz, CDCl_3_) δ 15.92, 99.74, 116.23, 117.38, 121.23, 122.31, 122.82, 123.98, 127.59, 128.04, 129.19, 129.26, 131.11, 142.79, 146.88, 150.39, 156.67, 160.21; HRMS (ESI) *m/z* calculated for C_20_H_15_O_3_ [M + H]^+^ 303.1016, found 303.1019. Purity by HPLC (0–18 min; 70% *n*-hexane/isopropanol): 98%.

Synthesis of 5-methyl-6-(2-oxopropyl)-3-phenyl-7*H*-furo[3,2-*g*]chromen-7-one (**8**):

This compound was synthesized following a previously described procedure [49]. Briefly, to a stirred solution of olefin **4** (158 mg, 0.5 mmol, 1 equiv.) in CH_3_CN (3.5 mL) and H_2_O (0.5 mL), Pd(OAc)_2_ (5.6 mg, 0.025 mmol, 5 mol %) and Dess-Martin periodinane (254 mg, 0.6 mmol, 1.2 equiv.) were added. The reaction mixture was warmed to 50 °C and stirred under an argon atmosphere overnight. The reaction mixture was then filtered through a small pad of Celite and washed with EtOAc, and the filtrate was concentrated. The residue was purified by column chromatography (EtOAc/*n*-hexane = 1/2, *v*/*v*, dry loading). White solid, yield: 40%. ^1^H-NMR (400 MHz, CDCl_3_) δ 2.32 (s, 3H, CH_2_COCH_3_), 2.44 (s, 3H, CH_3_), 3.88 (s, 2H, CH_2_COCH_3_), 7.44 (t, *J* = 7.4 Hz, 1H, Ar-H), 7.48–7.57 (m, 3H, 3 × Ar-H), 7.63 (app dd, *J* = 8.0, 1.0 Hz, 2H, 2 × Ar-H), 7.83 (s, 1H, Ar-H), 8.01 (s, 1H, Ar-H); ^13^C-NMR (101 MHz, CDCl_3_) δ 16.19, 30.15, 42.42, 100.13, 116.08, 117.27, 118.41, 122.51, 124.19, 127.77, 128.19, 129.41, 131.26, 143.01, 149.65, 150.81, 156.85, 161.93, 204.58; HRMS (ESI) *m/z* calculated for C_21_H_17_O_4_ [M + H]^+^ 333.1121, found 333.1127. Purity by HPLC (0–18 min; 70% *n*-hexane/isopropanol): 98%.

Synthesis of (*E*)-5-methyl-6-(2-(methylsulfonyl)vinyl)-3-phenyl-7*H*-furo[3,2-*g*]chromen-7-one (**9**):

To a solution of 5-methyl-3-phenyl-6-vinyl-7*H*-furo[3,2-*g*]chromen-7-one (**7**) (100 mg, 0.33 mmol, 1 equiv.) in DMSO (1 mL), H_2_O (0.5 mL) and NH_4_I (191 mg, 1.32 mmol, 4 equiv.) were added. The reaction mixture was stirred at 130 °C for 36 h. Then, it was cooled to room temperature, followed by slow addition of Na_2_S_2_O_3_ × 5H_2_O until the discoloration of mixture. Subsequently, H_2_O (20 mL) was added and the aqueous phase extracted with EtOAc (3 × 20 mL). The combined organic extracts were dried over Na_2_SO_4_, filtered, and evaporated under reduced pressure. The product was purified by column chromatography (EtOAc/*n*-hexane, 1/2, *v*/*v*). Yellow solid; yield: 64%; ^1^H-NMR (400 MHz, CDCl_3_) δ 2.63 (s, 3H, Ar-CH_3_), 3.14 (s, 3H, SO_2_CH_3_), 7.44–7.48 (m, 1H, Ar-CHCHSO_2_CH_3_), 7.52–7.57 (m, 4H, Ar-CHCHSO_2_CH_3_ and 3 × Ar-H), 7.61–7.65 (m, 3H, Ar-H), 7.87 (s, 1H, Ar-H), 8.11 (s, 1H, Ar-H); ^13^C-NMR (100 MHz, CDCl_3_): δ 15.08, 42.96, 60.48, 65.58, 100.25, 116.60, 116.89, 121.68, 122.48, 124.96, 127.66 (2C), 128.31, 129.38 (2C), 130.69, 143.40, 149.56, 150.40, 157.12, 161.99; HRMS (ESI) *m/z* calculated for C_21_H_17_O_5_S [M + H]^+^ 381.0791, found 381.0795.; Purity by HPLC (0–18 min; 70% *n*-hexane/isopropanol): 99%.

Synthesis of 6-(3-bromo-4,5-dihydroisoxazol-5-yl)-5-methyl-3-phenyl-7*H*-furo[3,2-*g*]chromen-7-one (**10**)

To a cooled (−15 °C) solution of 5-methyl-3-phenyl-6-vinyl-7*H*-furo[3,2-*g*]chromen-7-one (**7**) (145 mg, 0.48 mmol, 1 equiv.) and 1,1-dibromoformaldoxime (148 mg, 0.73 mmol, 1.5 equiv.) in DMF (10 mL), an aqueous solution of NaHCO_3_ (1 mL, 112 mg, 1.3 mmol, 2.7 equiv.) was added. The reaction was then warmed to room temperature and stirred for 5 h. Then, the solution was diluted with CH_2_Cl_2_ (20 mL) and washed with brine (20 mL). The organic extract was dried over Na_2_SO_4_, filtered, and the solvents removed under reduced pressure. The product was purified by column chromatography (EtOAc/*n*-hexane, 1/4, *v*/*v*) to yield pale yellow solid. Yield: 76%. ^1^H-NMR (400 MHz, CDCl_3_) δ 2.60 (s, 3H, Ar-CH_3_), 3.47 (dd, *J* = 17.1, 11.8 Hz, 1H, one H of CH_2_), 3.62 (dd, *J* = 17.1, 10.4 Hz, 1H, one H of CH_2_), 6.09 (dd, *J* = 11.8, 10.4 Hz, 1H, CH_2_CHO), 7.42–7.48 (m, 2H, Ar-H), 7.51–7.57 (m, 2H, Ar-H), 7.60–7.64 (m, 2H, Ar-H), 7.83 (s, 1H, Ar-H), 8.06 (s, 1H, Ar-H); ^13^C-NMR (101 MHz, CDCl_3_) δ 15.27, 46.05, 77.81, 100.01, 116.61, 116.75, 119.73, 122.36, 124.29, 127.60, 128.19, 129.33, 130.84, 137.27, 143.12, 150.88, 151.48, 157.17, 159.50; HRMS (ESI) *m/z* calculated for C_21_H_15_O_4_NBr [M + H]^+^ 424.0179, found 424.0179. Purity by HPLC (0–18 min; 70% *n*-hexane/isopropanol): 99%.

Synthesis of 5-methyl-3-phenyl-6-(2-(4,4,5,5-tetramethyl-1,3,2-dioxaborolan-2-yl)ethyl)-7*H*-furo[3,2-*g*]chromen-7-one (**11**):

5-Methyl-3-phenyl-6-vinyl-7*H*-furo[3,2-*g*]chromen-7-one (**7**) (0.09 mmol, 1 equiv.) was dissolved in 1,4-dioxane (2 mL) and then bis(pinacolato)diboron (1,5 equiv.), cesium fluoride (2,5 equiv.) and MeOH (5 equiv.) were added. The reaction proceeded at 100 °C for 12 h. The reaction mixture was then diluted with EtOAc (15 mL) and filtrated over silica. The filtrate was evaporated under reduced pressure and the product purified from the crude mixture by column chromatography (EtOAc/*n*-hexane, 1/9, gradient to 1/1, *v*/*v*) to yield yellow solid. Yield: 33%. ^1^H-NMR (400 MHz, CD_3_OD): δ 0.94 (t, *J* = 8.5 Hz, 2H, CH_2_), 1.13 (s, 12H, C(CH_3_)_2_), 2.48 (s, 3H, Ar-CH_3_), 2.68 (t, *J* = 8.5 Hz, 2H, CH_2_), 7.30–7.35 (m, 1H, Ar-H), 7.41–7.47 (m, 3H, Ar-H), 7.62–7.62 (m, 2H, Ar-H), 7.99 (s, 1H, Ar-H), 8.05 (s, 1H, Ar-H); ^13^C-NMR (100 MHz, CDCl_3_): δ 14.12, 17.77, 23.59 (4C), 29.40, 83.06, 85.22 (2C), 87.21, 102.00, 102.50, 105.99, 127.09 (2C), 127.58, 127.64, 128.75 (2C), 136.06, 141.54, 146.52, 152.51, 157.66, 161.81; HRMS (ESI) *m/z* calculated for C_26_H_28_O_5_B [M + H]^+^ 431.2024, found 431.2023.; Purity by HPLC (0–18 min; 95% *n*-hexane/isopropanol): 97%.

Synthesis of 3-(4-methyl-2-oxo-7-(2-oxo-2-phenylethoxy)-2*H*-chromen-3-yl)propanoic acid (**13**):

To a stirred solution of **12** (788 mg, 2 mmol, 1 equiv.) in dioxane (20 mL), HCl (1 M, 20 mL, 10 equiv.) was added. The reaction mixture was heated at reflux temperature for 2 h. Dioxane was then evaporated under reduced pressure, the precipitate that formed filtered off and washed with H_2_O. Yield: 94%. ^1^H-NMR (400 MHz, CDCl_3_) δ 2.44 (s, 3H, CH_3_), 2.67 (t, *J* = 7.6 Hz, 2H, CH_2_CH_2_COOH), 2.96 (t, *J* = 7.6 Hz, 2H, CH_2_CH_2_COOH), 5.38 (s, 2H, CH_2_), 6.78 (d, *J* = 2.6 Hz, 1H, Ar-H), 6.95 (dd, *J* = 8.9, 2.6 Hz, 1H, Ar-H), 7.49–7.60 (m, 3H, 3 × Ar-H), 7.66 (t, *J* = 7.4 Hz, 1H, Ar-H), 7.82–8.07 (m, 2H, 2 × Ar-H); HRMS (ESI) *m/z* calculated for C_21_H_17_O_6_ [M − H]^−^ 365.1031, found 365.1030.

Synthesis of 3-(4-methyl-2-oxo-7-(2-oxo-2-phenylethoxy)-2*H*-chromen-3-yl)propanal (**14**):

To a suspension of **13** (366 mg, 1 mmol, 1 equiv.) in toluene (20 mL), dried over 3 Å molecular sieves, a catalytic amount of anhydrous DMF (5 drops) and SOCl_2_ (218 μL, 357 mg, 3 mmol, 3 equiv.) were added under argon. The reaction mixture was stirred at room temperature for 17 h and then the volatiles were evaporated to obtain a white solid that was dried under vacuum for 15 min to remove SOCl_2_. Toluene (20 mL), dried over 3 Å molecular sieves, was added to the dried solid (under argon), followed by the addition of 10% Pd/BaSO_4_ (72 mg, 30% [*w*/*w*]). The reaction mixture was heated to 100 °C and stirred under a stream of hydrogen (1 atm) for 2 h. The reaction mixture was then evaporated to dryness and the compound was purified by column chromatography (EtOAc/*n*-hexane, 1/1, *v*/*v*, dry loading). White solid, yield: 59%. ^1^H-NMR (400 MHz, CDCl_3_) δ 2.43 (s, 3H, CH_3_), 2.77 (t, *J* = 7.4 Hz, 2H, CH_2_CH_2_CHO), 2.94 (t, *J* = 7.4 Hz, 2H, CH_2_CH_2_CHO), 5.38 (s, 2H, CH_2_), 6.78 (d, *J* = 2.6 Hz, 1H, Ar-H), 6.95 (dd, *J* = 8.9, 2.6 Hz, 1H, Ar-H), 7.50–7.59 (m, 3H, 3 × Ar-H), 7.66 (t, *J* = 7.4 Hz, 1H, Ar-H), 7.04–8.06 (m, 2H, 2 × Ar-H), 9.83 (t, *J* = 1.0 Hz, 1H, CH_2_CH_2_CHO); HRMS (ESI) *m/z* calculated for C_21_H_19_O_5_ [M + H]^+^ 351.1227, found 351.1221.

Synthesis of 3-(5-methyl-7-oxo-3-phenyl-7*H*-furo[3,2-*g*]chromen-6-yl)propanal (**15**):

To a suspension of **14** (519 mg, 1.48 mmol, 1 equiv.) in propan-2-ol (35 mL), an aqueous solution of NaOH (14.8 mL, 1 M, 10 equiv.) was added. The reaction mixture was stirred at 60 °C for 15 min. After the reaction was complete (monitored by TLC), the resulting red solution was acidified with 1 M HCl (15 mL) to get a yellow precipitate. The reaction mixture was evaporated under reduced pressure. H_2_O (50 mL) was added to the dry residue, which was extracted with CH_2_Cl_2_ (2 × 50 mL), the combined organic extracts were washed with brine (100 mL), dried over anhydrous Na_2_SO_4_, filtered, and evaporated under reduced pressure. The compound was purified by column chromatography (EtOAc/*n*-hexane, 1/2, *v*/*v*, dry loading). White solid, yield: 61%. ^1^H-NMR (400 MHz, CDCl_3_) δ 2.55 (s, 3H, CH_3_), 2.82 (t, *J* = 7.4 Hz, 2H, CH_2_CH_2_CHO), 3.01 (t, *J* = 7.4 Hz, 2H, CH_2_CH_2_CHO), 7.47–7.41 (m, 1H, Ar-H), 7.49 (s, 1H, Ar-H), 7.50–7.57 (m, 2H, 2 × Ar-H), 7.60–7.67 (m, 2H, 2 × Ar-H), 7.83 (s, 1H, Ar-H), 8.00 (s, 1H, Ar-H), 9.86 (t, *J* = 1.0 Hz, 1H, CH_2_CH_2_CHO); ^13^C-NMR (101 MHz, CDCl_3_) δ 15.67, 20.85, 42.53, 99.97, 115.99, 117.34, 122.46, 122.98, 124.09, 127.72, 128.18, 129.39, 131.24, 142.94, 147.70, 150.56, 156.58, 161.72, 201.40; HRMS (ESI) *m/z* calculated for C_21_H_17_O_4_ [M + H]^+^ 333.1121, found 333.1116. Purity by HPLC (0–18 min; 70% *n*-hexane/isopropanol): 87%.

Synthesis of 3-(5-methyl-7-oxo-3-phenyl-7*H*-furo[3,2-*g*]chromen-6-yl)acrylaldehyde (**16**):

To a solution of the aldehyde **15** (53 mg, 0.16 mmol, 1 equiv.) in a mixture of dioxane (1.5 mL) and H_2_O (20 µL), SeO_2_ (35 mg, 0.32 mmol, 2 equiv.) was added and the reaction mixture was irradiated in a microwave reactor at 150 °C (250 W) for 1 h. The reaction mixture was then evaporated to dryness and the compound was purified by column chromatography (EtOAc/*n*-hexane, 1/2, *v/v*, dry loading). White solid, yield: 10%. ^1^H-NMR (400 MHz, CDCl_3_) δ 2.74 (s, 3H, CH_3_), 7.32 (dd, *J* = 15.8, 7.5 Hz, 1H, CHCHCHO), 7.46 (t, *J* = 7.4 Hz, 1H, Ar-H), 7.51 (s, 1H, Ar-H), 7.52–7.59 (m, 2H, 2 × Ar-H), 7.61–7.69 (m, 3H, 2 × Ar-H and CHCHCHO), 7.85 (s, 1H, Ar-H), 8.14 (s, 1H, Ar-H), 9.74 (d, *J* = 7.5 Hz, 1H, CHCHCHO); ^13^C-NMR (101 MHz, CDCl_3_) δ 16.21, 100.29, 116.93, 117.39, 118.27, 122.59, 124.84, 127.79, 128.42, 129.49, 130.86, 134.87, 143.45, 143.55, 151.20, 152.79, 157.88, 158.95, 194.53; HRMS (ESI) *m/z* calculated for C_21_H_15_O_4_ [M + H]^+^ 331.0965, found 331.0979. Purity by HPLC (0–18 min; 70% *n*-hexane/isopropanol): 97%.

Synthesis of 5-methyl-*N*-((*S*)-1-((*R*)-2-methyloxiran-2-yl)-l-oxopropan-2-yl)-7-oxo-3-phenyl-7*H*-furo[3,2-*g*]chromene-6-carboxamide (**20**):

To a cooled (0 °C) solution of compound **17** (160 mg, 0.50 mmol, 1 equiv.) in DMF (4 mL), HATU (285 mg, 0.75 mmol, 1.5 equiv.) and HOBt hydrate (115 mg, 0.75 mmol, 1.5 equiv.) were added. In a separate round-bottom flask, compound **18** (115 mg, 0.50 mmol, 1 equiv.) was dissolved in CH_2_Cl_2_ (3 mL) at 0 °C, followed by the addition of TFA (3 mL). After 30 min of stirring at 0 °C, the volatiles were evaporated under reduced pressure thoroughly, the residue was dissolved in CH_2_Cl_2_ and slowly added to the mixture containing compound **17** at 0 °C. After 5 min, DIPEA (348 µL, 285 mg, 2.0 mmol, 4 equiv.) was added and the reaction mixture stirred at room temperature for 24 h. Then, the solvent was evaporated and the product purified by column chromatography (EtOAc/*n*-hexane, 1/1, *v*/*v*, dry loading) without additional work-up. Off-white solid, yield: 16%. ^1^H-NMR (400 MHz, CDCl_3_) δ 1.44 (d, *J* = 7.0 Hz, 3H, CHCH_3_), 1.57 (s, 3H, CH_3_), 2.75 (s, 3H, Ar-CH_3_), 2.96 (d, *J* = 5.0 Hz, 1H, one H of oxirane CH_2_), 3.39 (app d, *J* = 5.0 Hz, 1H, one H of oxirane CH_2_), 4.72 (p, *J* = 6.7 Hz, 1H, CHCH_3_), 7.42–7.48 (m, 1H, Ar-H), 7.51 (s, 1H, Ar-H), 7.52–7.57 (m, 2H, Ar-H), 7.59–7.65 (m, 3H, CONH + Ar-H), 7.85 (s, 1H, Ar-H), 8.13 (s, 1H, Ar-H), ^13^C-NMR (101 MHz, CDCl_3_) δ 16.87, 16.92 (2C), 48.68, 52.70, 59.15, 100.04, 116.76, 117.32, 118.53, 122.43, 124.69, 127.63, 128.23, 129.33, 130.71, 143.27, 150.83, 155.64, 157.64, 160.02, 163.86, 208.01; HRMS (ESI) *m/z* calculated for C_25_H_22_O_6_N [M + H]^+^ 432.1442, found 432.1438. Purity by HPLC (0–18 min; 70% *n*-hexane/isopropanol): 96%.

Synthesis of 5-methyl-*N*-((*S*)-1-((*R*)-2-methyloxiran-2-yl)-l-oxo-3-phenylpropan-2-yl)-7-oxo-3-phenyl-7*H*-furo[3,2-*g*]chromene-6-carboxamide (**21**):

To a cooled (0 °C) solution of compound **17** (28 mg, 0.087 mmol, 1 equiv.) in CH_2_Cl_2_ (4 mL), HATU (40 mg, 0.11 mmol, 1.2 equiv.) and HOBt hydrate (17 mg, 0.11 mmol, 1.2 equiv.) were added. In a separate round-bottom flask, compound **19** (27 mg, 0.087 mmol, 1 equiv.) was dissolved in CH_2_Cl_2_ (2 mL) at 0 °C, followed by the addition of TFA (2 mL). After 30 min of stirring at 0 °C, the volatiles were evaporated under reduced pressure thoroughly, the residue was dissolved in CH_2_Cl_2_ and slowly added to the mixture containing compound **17** at 0 °C. After 5 min, DIPEA (58 µL, 45 mg, 0.35 mmol, 4 equiv.) was added and the reaction mixture stirred at room temperature for 24 h. Then, the solvent was evaporated and the product purified by column chromatography (EtOAc/*n*-hexane, 1/2, *v*/*v*, dry loading) without additional work-up. Off-white solid, yield: 11%. ^1^H-NMR (400 MHz, CDCl_3_) δ 1.55 (s, 3H, CH_3_), 2.65 (s, 3H, Ar-CH_3_), 2.88 (dd, *J* = 13.7, 8.7 Hz, 1H, one H of oxirane CH_2_), 2.98 (d, *J* = 5.0 Hz, 1H, one H of oxirane CH_2_), 3.27 (dd, *J* = 13.7, 4.8 Hz, 1H, one H of CHCH_2_Ph), 3.47 (dd, *J* = 5.0, 0.5 Hz, 1H, one H of CHCH_2_Ph), 4.99 (symm m, 1H, CHCH_3_), 7.27–7.35 (m, 5H, Ar-H), 7.42–7.47 (m, 1H, Ar-H), 7.50 (d, *J* = 0.4 Hz, 1H, Ar-H), 7.52–7.56 (m, 2H, Ar-H), 7.59–7.63 (m, 2H, Ar-H), 7.84 (s, 1H, Ar-H), 7.85 (br d, *J* = 6.7 Hz, 1H, CONH), 8.11 (s, 1H, Ar-H); ^13^C-NMR was not recorded due to insufficient amount of the final product; HRMS (ESI) *m/z* calculated for C_31_H_26_O_6_N [M + H]^+^ 508.1755, found 508.1755. Purity by HPLC (0–18 min; 95% *n*-hexane/isopropanol): 97%.

Synthesis of 3-bromo-*N*-((5-methyl-7-oxo-3-phenyl-7*H*-furo[3,2-*g*]chromen-6-yl)methyl)propanamide (**23**):

To a cooled (0 °C) solution of compound **22** (92 mg, 0.3 mmol, 1 equiv.) in CH_2_Cl_2_ (10 mL), K_2_CO_3_ (50 mg, 0.36 mmol, 1.2 equiv.) was added. After 5 min, 3-bromopropanoyl chloride (36 µL, 62 mg, 0.36 mmol, 1.2 equiv.) was added drop-wise at 0 °C. The reaction mixture was then stirred at room temperature for 3 h. The reaction was quenched by the addition of H_2_O (20 mL) and the mixture was extracted with EtOAc (3 × 50 mL). The combined organic extracts were dried over Na_2_SO_4_, filtered, and evaporated under reduced pressure. The product was purified by column chromatography (EtOAc/*n*-hexane, 1/2, *v*/*v*). White solid, yield: 90%. ^1^H-NMR (400 MHz, CDCl_3_) δ 2.73 (s, 3H, CH_3_), 2,74 (t, *J* = 6.7 Hz, 2H, COCH_2_), 3.61 (t, *J* = 6.7 Hz, 2H, CH_2_Br), 4.52 (d, *J* = 6.2 Hz, 2H, Ar-CH_2_NH), 7.42–7.47 (m, 1H, Ar-H), 7.51–7.56 (m, 3H, Ar-H), 7.61–7.64 (m, 2H, Ar-H), 7.83 (s, 1H, Ar-H), 8.07 (s, 1H, Ar-H); ^13^C-NMR (101 MHz, CDCl_3_) δ 15.59, 27.13, 36.69, 39.51, 100.01, 116.75, 117.07, 120.68, 122.43, 124.31, 127.61 (2C), 128.15, 129.32 (2C), 130.96, 142.99, 149.61, 150.66, 156.81, 162.37, 169.52; HRMS (m/z) (ESI): calculated for C_22_H_19_O_4_NBr [M + H]^+^ 440.0492, found: 440.0490; Purity by HPLC (0–18 min; 70% *n*-hexane/isopropanol): 96%.

Synthesis of 1-((5-methyl-7-oxo-3-phenyl-7*H*-furo[3,2-*g*]chromen-6-yl)methyl)azetidin-2-one (**24**):

To a cooled (0 °C) solution of compound **23** (66 mg, 0.15 mmol, 1 equiv.) in DMF (15 mL), NaO*t*Bu (16 mg, 0.17 mmol, 1.1 equiv.) was added. The reaction mixture was stirred at room temperature for 24 h. The reaction was quenched by the addition of H_2_O (20 mL) and the mixture was extracted with EtOAc (3 × 50 mL). The combined organic extracts were dried over Na_2_SO_4_, filtered, and evaporated under reduced pressure. The product was purified by column chromatography (EtOAc/*n*-hexane, 1/2, *v*/*v*). White solid, yield: 87%. ^1^H-NMR (400 MHz, CDCl_3_) δ 2.73 (s, 3H, CH_3_), 2.74 (t, *J* = 6.4 Hz, 2H, azetidin-2-one-CH_2_), 3.61 (t, *J* = 6.4 Hz, 2H, azetidin-2-one-CH_2_), 4.52 (s, 2H, Ar-CH_2_N), 7.42–7.47 (m, 1H, Ar-H), 7.52 (s, 1H, Ar-H), 7.53–7.56 (m, 2H, Ar-H), 7.61–7.65 (m, 2H, Ar-H), 7.84 (s, 1H, Ar-H), 8.07 (s, 1H, Ar-H); ^13^C-NMR (101 MHz, CDCl_3_) δ 29.72, 31.95, 37.04, 38.55, 102.72, 112.28, 115.88, 116.54, 116.80, 122.43, 122.51, 127.62 (2C), 128.16, 128.74, 129.33 (2C), 132.78, 143.00, 156.80, 157.68, 178.20; HRMS (m/z) (ESI): calculated for C_22_H_18_O_4_N [M + H]^+^ 360.1230, found: 360.1222; Purity by HPLC (0–18 min; 70% *n*-hexane/isopropanol): 87%.

### 3.3. Residual Activity Measurements

The screening of compounds was performed at 1 μM final concentrations in the assay buffer (0.01% SDS, 50 mM Tris-HCl, 0.5 mM EDTA, pH 7.4). Stock solutions of compounds were prepared in DMSO. To 50 μL of each compound, 25 μL of 0.8 nM human iCP or human cCP (both from Boston Biochem, Inc., Cambridge, MA, USA) was added. After 30 min incubation at 37 °C, the reaction was initiated by the addition of 25 μL of 100 μM relevant fluorogenic substrate: acetyl-Nle-Pro-Nle-Asp-AMC (Ac-nLPnLD-AMC, [Bachem, Bubendorf, Switzerland]) for β1, acetyl-Pro-Ala-Leu-7-amino-4-methylcoumarin (Ac-PAL-AMC, [Boston Biochem, Inc., Cambridge, MA, USA]) for β1i, *t*-butyloxycarbonyl-Leu-Arg-Arg-7-amino-4-methylcoumarin (Boc-LRR-AMC, [Bachem, Bubendorf, Switzerland]) for β2 and β2i, succinyl-Leu-Leu-Val-Tyr-7-amino-4-methylcoumarin (Suc-LLVY-AMC [Bachem, Bubendorf, Switzerland]) for β5 and β5i. The reaction progress was recorded on the BioTek Synergy HT microplate reader by monitoring fluorescence at 460 nm (λ_ex_ = 360 nm) for 90 min at 37 °C. The initial linear ranges were used to calculate the velocity and to determine the residual activity.

In the case of the β1, β1i, β2, and β2i activity inhibition determination, the assay buffer was modified; SDS was replaced with the proteasomal activator PA28α (Boston Biochem, Inc., Cambridge, MA, USA).

### 3.4. Molecular Modelling

Compounds were prepared for docking using LigPrep (Schrödinger Suite 2020-2, Schrödinger, LLC, New York, NY, USA, 2020) to account for all possible tautomers and ionization states at pH 7.0 ± 2.0. The X-ray structure (PDB: 5M2B, [43]) of yeast 20S proteasome with human β5i and β1 subunits in complex with noncovalent inhibitor Ro19 was used for docking. The binding site is defined by the chain K (β5i) and neighbouring L (β1), so all other chains were removed. Protein Preparation Wizard [50] was used to add hydrogen atoms, protonate residues at pH 7, refine the H-bond network and to perform a restrained minimization. The receptor’s grid box required for docking calculations was centred on the corresponding co-crystallized ligand. Noncovalent docking was performed using Glide [51], with the following parameters: XP (extra precision), flexible ligand sampling, perform postdocking minimization. Covalent docking was performed with CovDock program [52] using the pose prediction mode with default setup and Thr1 defined as the reactive residue. Nucleophilic addition to a double bond (oxathiazolone) was selected as the reaction.

## 4. Conclusions

Here, we showed that the introduction of 12 new electrophilic warheads at position 3 of the psoralen ring led to compounds with abrogated inhibition of the iCP (especially β5i subunit). As already described in the Introduction, it is imperative that the initial non-covalent binding of a given compound is followed by the positioning of the electrophilic ‘warhead’ near the desired nucleophilic amino-acid residue of the protein to achieve covalent interaction. Poor inhibition results were in our cases most probably due to the mispositioning of the electrophilic carbon and the catalytic Thr1O*^γ^* (Figure 7). The oxathiazolone thus remains the optimal electrophilic moiety for this compound class. Despite somewhat disappointing results, the obtained data will help steer our future research in the field of psoralen-based iCP inhibitors, e.g., when designing inhibitors which simultaneously inhibit two iCP subunits as it was established that simultaneous inhibition of β1i and β5i is necessary to achieve significant anti-inflammatory effects.

## Figures and Tables

**Table 1:** Inhibitory potencies of compounds against all catalytically active subunits (β5i, β2i, and β1i) of the iCP and against all catalytically active subunits (β5, β2, β1) of the human cCP. In the assays, the following substrates were used: Suc-LLVY-AMC for β5i and β5; Boc-LRR-AMC for β2i and β2; Ac-PAL-AMC for β1i; Ac-nLPnLD-AMC for β1.

Cpd	β5i (RA [%]) ^1^	β2i (RA [%]) ^1^	β1i (RA [%]) ^1^	β5 (RA [%]) ^1^	β2 (RA [%]) ^1^	β1 (RA [%]) ^1^
**4**	78 ± 5	100 ± 0	95 ± 24	80 ± 21	82 ± 7	88 ± 0
**7**	76 ± 3	100 ± 0	87 ± 15	81 ± 18	86 ± 7	88 ± 5
**8**	70 ± 0	100 ± 0	90 ± 21	78 ± 21	87 ± 7	89 ± 2
**9**	69 ± 13	109 ± 3	76 ± 7	72 ± 18	90 ± 2	99 ± 5
**10**	62 ± 5	102 ± 2	87 ± 19	79 ± 20	86 ± 2	90 ± 3
**11**	76 ± 12	109 ± 14	72 ± 8	66 ± 21	89 ± 4	87 ± 2
**15**	71 ± 1	103 ± 4	94 ± 16	76 ± 20	87 ± 3	97 ± 2
**16**	65 ± 3	107 ± 3	92 ± 21	77 ± 19	83 ± 4	83 ± 2
**20**	78 ± 0	88 ± 0	83 ± 18	76 ± 17	81 ± 6	79 ± 5
**21**	76 ± 1	90 ± 0	81 ± 10	79 ± 14	80 ± 4	86 ± 4
**23**	74 ± 11	113 ± 7	72 ± 1	72 ± 12	89 ± 6	92 ± 4
**24**	77 ± 7	109 ± 7	74 ± 5	63 ± 26	88 ± 5	89 ± 7
**carf.**	3 ± 1	1 ± 1	1 ± 1	0 ± 0	16 ± 6	2 ± 2
**‘42’**	5 ± 2	102 ± 5	97 ± 8	52 ± 4	99 ± 2	99 ± 8

^1^ RA values are means from at least three independent determinations. Ac-PAL-AMC, acetyl-Pro-Ala-Leu-7-amino-4-methylcoumarin; Ac-nLPnLD-AMC, acetyl-Nle-Pro-Nle-Asp-AMC; Boc-LRR-AMC, *t*-butyloxycarbonyl-Leu-Arg-Arg-7-amino-4-methylcoumarin; Suc-LLVY-AMC, succinyl-Leu-Leu-Val-Tyr-7-amino-4-methylcoumarin. carf.: carfilzomib.

## Data Availability

The data presented in this study are available on request from the corresponding author.

## Sample Availability

Samples of all compounds, except compound **21** are available from the authors.

## References

[1] Rousseau A., Bertolotti A. (2018). Regulation of proteasome assembly and activity in health and disease. Nat. Rev. Mol. Cell Biol..

[2] Hershko A., Ciechanover A. (1998). The ubiquitin system. Annu. Rev. Biochem..

[3] DeMartino G.N., Gillette T.G. (2007). Proteasomes: Machines for all reasons. Cell.

[4] Collins G.A., Goldberg A.L. (2017). The logic of the 26S proteasome. Cell.

[5] Goldberg A.L. (2007). Functions of the proteasome: From protein degradation and immune surveillance to cancer therapy. Biochem. Soc. Trans..

[6] Gallastegui N., Groll M. (2010). The 26S proteasome: Assembly and function of a destructive machine. Trends Biochem. Sci..

[7] Thibaudeau T.A., Smith D.M. (2019). A practical review of proteasome pharmacology. Pharmacol. Rev..

[8] Groll M., Ditzel L., Lowe J., Stock D., Bochtler M., Bartunikt H.D., Huber R. (1997). Structure of 20S proteasome from yeast at 2.4 A resolution. Nature.

[9] Arendt C.S., Hochstrasser M. (1997). Identification of the Yeast 20S Proteasome Catalytic Centers and Subunit Interactions Required for Active-Site Formation. Proc. Natl. Acad. Sci. USA.

[10] Budenholzer L., Cheng C.L., Li Y., Hochstrasser M. (2017). Proteasome structure and assembly. J. Mol. Biol..

[11] Murata S., Sasaki K., Kishimoto T., Niwa S.-I., Hayashi H., Takahama Y., Tanaka K. (2007). Regulation of CD8^+^ T cell development by thymus-specific proteasomes. Science.

[12] Groettrup M., Kirk C.J., Basler M. (2010). Proteasomes in immune cells: More than peptide producers?. Nat. Rev. Immunol..

[13] Tanaka K. (1994). Role of proteasomes modified by interferon-γ in antigen processing. J. Leukoc. Biol..

[14] Aki M., Shimbara N., Takashina M., Akiyama K., Kagawa S., Tamura T., Tanahashi N., Yoshimura T., Tanaka K., Ichihara A. (1994). Interferon-γ induces different subunit organizations and functional diversity of proteasomes. J. Biochem..

[15] Nandi D., Jiang H., Monaco J.J. (1996). Identification of MECL-1 (LMP-10) as the third IFN-gamma-inducible proteasome subunit. J. Immunol..

[16] Basler M., Kirk C.J., Groettrup M. (2013). The immunoproteasome in antigen processing and other immunological functions. Curr. Opin. Immunol..

[17] Almond J.B., Cohen G.M. (2002). The proteasome: A novel target for cancer chemotherapy. Leukemia.

[18] Kuhn D.J., Orlowski R.Z. (2012). The immunoproteasome as a target in hematologic malignancies. Semin. Hematol..

[19] Ito S. (2020). Proteasome inhibitors for the treatment of multiple myeloma. Cancers.

[20] Verbrugge E.E., Scheper R.J., Lems W.F., de Gruijl T.D., Jansen G. (2015). Proteasome inhibitors as experimental therapeutics of autoimmune diseases. Arthritis Res. Ther..

[21] Basler M., Mundt S., Bitzer A., Schmidt C., Groettrup M. (2015). The immunoproteasome: A novel drug target for autoimmune diseases. Clin. Exp. Rheumatol..

[22] Zhang C., Zhu H., Shao J., He R., Xi J., Zhuang R., Zhang J. (2020). Immunoproteasome-selective inhibitors: The future of autoimmune diseases?. Future Med. Chem..

[23] Limanaqi F., Biagioni F., Gaglione A., Busceti C.L., Fornai F. (2019). A sentinel in the crosstalk between the nervous and immune system: The (immuno)-proteasome. Front. Immunol..

[24] Huber E.M., Groll M. (2012). Inhibitors for the immuno- and constitutive proteasome: Current and future trends in drug development. Angew. Chem. Int. Ed..

[25] Cromm P.M., Crews C.M. (2017). The proteasome in modern drug discovery: Second life of a highly valuable drug target. ACS Cent. Sci..

[26] Richy N., Sarraf D., Maréchal X., Janmamode N., Le Guével R., Genin E., Reboud-Ravaux M., Vidal J. (2018). Structure-based design of human immuno- and constitutive proteasomes inhibitors. Eur. J. Med. Chem..

[27] Sherman D.J., Li J. (2020). Proteasome inhibitors: Harnessing proteostasis to combat disease. Molecules.

[28] Muchamuel T., Basler M., Aujay M.A., Suzuki E., Kalim K.W., Lauer C., Sylvain C., Ring E.R., Shields J., Jiang J. (2009). A selective inhibitor of the immunoproteasome subunit LMP7 blocks cytokine production and attenuates progression of experimental arthritis. Nat. Med..

[29] Johnson H.W.B., Lowe E., Anderl J.L., Fan A., Muchamuel T., Bowers S., Moebius D.C., Kirk C., McMinn D.L. (2018). Required immunoproteasome subunit inhibition profile for anti-inflammatory efficacy and clinical candidate KZR-616 ((2*S*,3*R*)-*N*-((*S*)-3-(cyclopent-1-en-1-yl)-1-((*R*)-2-methyloxiran-2-yl)-1-oxopropan-2-yl)-3-hydroxy-3-(4-methoxyphenyl)-2-((*S*)-2-(2-morpholinoacetamido)propanamido)propenamide). J. Med. Chem..

[30] Basler M., Lindstrom M.M., LaStant J.J., Bradshaw J.M., Owens T.D., Schmidt C., Meurits E., Tsu C., Overkleeft H.S., Kirk C.J. (2018). Co-inhibition of immunoproteasome subunits LMP2 and LMP7 is required to block autoimmunity. EMBO Rep..

[31] Ettari R., Zappalà M., Grasso S., Musolino C., Innao V., Allegra A. (2018). Immunoproteasome-selective and non-selective inhibitors: A promising approach for the treatment of multiple myeloma. Pharmacol. Ther..

[32] Xi J., Zhuang R., Kong L., He R., Zhu H., Zhang J. (2019). Immunoproteasome-selective inhibitors: An overview of recent developments as potential drugs for hematologic malignancies and autoimmune diseases. Eur. J. Med. Chem..

[33] Ladi E., Everett C., Stivala C.E., Daniels B.E., Durk M.R., Harris S.F., Huestis M.P., Purkey H.E., Staben S.T., Augustin M. (2019). Design and evaluation of highly selective human immunoproteasome inhibitors reveal a compensatory process that preserves immune cell viability. J. Med. Chem..

[34] Karreci E.S., Fan H., Uehara M., Mihali A.B., Singh P.K., Kurdi A.T., Solhjou Z., Riella L.V., Ghobrial I., Laragione T. (2016). Brief treatment with a highly selective immunoproteasome inhibitor promotes long-term cardiac allograft acceptance in mice. Proc. Natl. Acad. Sci. USA.

[35] Ogorevc E., Schiffrer E.S., Sosič I., Gobec S. (2018). A patent review of immunoproteasome inhibitors. Expert Opin. Ther. Pat..

[36] Tan C.R.C., Abdul-Majeed S., Cael B., Barta S.K. (2019). Clinical pharmacokinetics and pharmacodynamics of bortezomib. Clin. Pharmacokinet..

[37] Yang J., Wang Z., Fang Y., Jiang J., Zhao F., Wong H., Bennett M.K., Molineaux C.J., Kirk C.J. (2011). Pharmacokinetics, pharmacodynamics, metabolism, distribution, and excretion of carfilzomib in rats. Drug Metab. Dispos..

[38] Wang Z., Yang J., Kirk C., Fang Y., Alsina M., Badros A., Papadopoulos K., Wong A., Woo T., Bomba D. (2013). Clinical pharmacokinetics, metabolism, and drug-drug interaction of carfilzomib. Drug Metab. Dispos..

[39] Sosič I., Gobec M., Brus B., Knez D., Živec M., Konc J., Lešnik S., Ogrizek M., Obreza A., Žigon D. (2016). Nonpeptidic selective inhibitors of the chymotrypsin-like (β5i) subunit of the immunoproteasome. Angew. Chem. Int. Ed..

[40] Schiffrer E.S., Sosič I., Šterman A., Mravljak J., Raščan I.M., Gobec S., Gobec M. (2019). A focused structure-activity relationship study of psoralen-based immunoproteasome inhibitors. Med. Chem. Commun..

[41] Fan H., Angelo N.G., Warren J.D., Nathan C.F., Lin G. (2014). Oxathiazolones selectively inhibit the human immunoproteasome over the constitutive proteasome. ACS Med. Chem. Lett..

[42] Kasam V., Lee N.-R., Kim K.-B., Zhan C.-G. (2014). Selective immunoproteasome inhibitors with non-peptide scaffolds identified from structure-based virtual screening. Bioorg. Med. Chem. Lett..

[43] Cui H., Baur R., Le Chapelain C., Dubiella C., Heinemeyer W., Huber E.M., Groll M. (2017). Structural elucidation of a nonpeptidic inhibitor specific for the human immunoproteasome. ChemBioChem.

[44] Scarpino A., Bajusz D., Proj M., Gobec M., Sosič I., Gobec S., Ferenczy G.G., Keseru G.M. (2019). Discovery of immunoproteasome inhibitors using large-scale covalent virtual screening. Molecules.

[45] Singh J., Petter R.C., Baillie T.A., Whitty A. (2011). The resurgence of covalent drugs. Nat. Rev. Drug Discov..

[46] Pinto A., Tamborini L., Cullia G., Conti P., De Micheli C. (2016). Inspired by nature: The 3-halo-4,5-dihydroisoxazole moiety as a novel molecular warhead for the design of covalent inhibitors. ChemMedChem.

[47] Huber E.M., de Bruin G., Heinemeyer W., Soriano G.P., Overkleeft H.S., Groll M. (2015). Systematic analyses of substrate preferences of 20S proteasomes using peptidic epoxyketone inhibitors. J. Am. Chem. Soc..

[48] Zhou H.J., Aujay M.A., Bennett M.K., Dajee M., Demo S.D., Fang Y., Ho M.N., Jiang J., Kirk C.J., Laidig G.J. (2009). Design and synthesis of an orally bioavailable and selective peptide epoxyketone proteasome inhibitor (PR-047). J. Med. Chem..

[49] Chaudhari D.A., Fernandes R.A. (2016). Hypervalent iodine as a terminal oxidant in wacker-type oxidation of terminal olefins to methyl ketones. J. Org. Chem..

[50] Madhavi Sastry G., Adzhigirey M., Day T., Annabhimoju R., Sherman W. (2013). Protein and ligand preparation: Parameters, protocols, and influence on virtual screening enrichments. J. Comput. Aided. Mol. Des..

[51] Friesner R.A., Murphy R.B., Repasky M.P., Frye L.L., Greenwood J.R., Halgren T.A., Sanschagrin P.C., Mainz D.T. (2006). Extra precision glide: Docking and scoring incorporating a model of hydrophobic enclosure for protein-ligand complexes. J. Med. Chem..

[52] Zhu K., Borrelli K.W., Greenwood J.R., Day T., Abel R., Farid R.S., Harder E. (2014). Docking covalent inhibitors: A parameter free approach to pose prediction and scoring. J. Chem. Inf. Model..

